# Study on the Compressive Strength and Reaction Mechanism of Alkali-Activated Geopolymer Materials Using Coal Gangue and Ground Granulated Blast Furnace Slag

**DOI:** 10.3390/ma17153659

**Published:** 2024-07-24

**Authors:** Xiaoping Wang, Feng Liu, Lijuan Li, Weizhi Chen, Xinhe Cong, Ting Yu, Baifa Zhang

**Affiliations:** 1 School of Civil and Transportation Engineering, Guangdong University of Technology, Guangzhou 510006, China; 1122109004@mail2.gdut.edu.cn (X.W.);; 2 School of Architecture and Engineering, Huangshan University, Huangshan 245041, China; 3 School of Environmental Science and Engineering, Guangdong University of Technology, Guangzhou 510006, China

**Keywords:** alkali-activated materials, coal gangue, ground granulated blast furnace slag, mechanical properties, microstructure

## Abstract

By reutilizing industrial byproducts, inorganic cementitious alkali-activated materials (AAMs) contribute to reduced energy consumption and carbon dioxide (CO_2_) emissions. In this study, coal gangue (CG) blended with ground granulated blast furnace slag (GGBFS) was used to prepare AAMs. The research focused on analyzing the effects of the GGBFS content and alkali activator (i.e., Na_2_O mass ratio and alkali modulus [SiO_2_/Na_2_O]) on the mechanical properties and microstructures of the AAMs. Through a series of spectroscopic and microscopic tests, the results showed that the GGBFS content had a significant influence on AAM compressive strength and paste fluidity; the optimal replacement of CG by GGBFS was 40–50%, and the optimal Na_2_O mass ratio and alkali modulus were 7% and 1.3, respectively. AAMs with a 50% GGBFS content exhibited a compact microstructure with a 28 d compressive strength of 54.59 MPa. Increasing the Na_2_O mass ratio from 6% to 8% promoted the hardening process and facilitated the formation of AAM gels; however, a 9% Na_2_O mass ratio inhibited the condensation of SiO_4_ and AlO_4_ ions, which decreased the compressive strength. Increasing the alkali modulus facilitated geopolymerization, which increased the compressive strength. Microscopic analysis showed that pore size and volume increased due to lower Na_2_O concentrations or alkali modulus. The results provide an experimental and theoretical basis for the large-scale utilization of AAMs in construction.

## 1. Introduction

Ordinary Portland cement (OPC) is an essential component of concrete. Generally, the production of 1 ton of OPC generates 0.8–1.0 tons of carbon dioxide (CO_2_), and as the global population increases, the demand for OPC increases. The cement industry is estimated to account for 7–8% of global CO_2_ emissions, which is challenging for human societies [1,2,3]. It is predicted that the production of OPC will reach 5 billion tons in 2030, and the projected emission of CO_2_ could be as high as 4.7 billion tons [4]. The global consensus on CO_2_ reduction and emissions has promoted research on low-carbon cementitious materials as alternatives to OPC [5,6].

In recent years, alkali-activated materials (AAMs) have been extensively used in construction [7]. AAMs are the product of polymerization reactions between aluminosilicate precursors and alkali activators [8,9,10]. They exhibit high compressive strength [11], good thermal properties [12], and corrosion resistance [13,14]. The production of AAMs consumes less energy and produces lower CO_2_ emissions [15,16]. As recorded, AAMs show an 80% reduction in CO_2_ emissions compared to OPC [17]. Therefore, AAMs are considered an alternative material to OPC due to their attractive physical properties and low CO_2_ emissions. Increasing numbers of byproducts have been used as precursor materials for preparing AAMs, such as metakaolin (MK) [18], fly ash (FA) [19], and ground granulated blast furnace slag (GGBFS) [20]. Furthermore, other aluminosilicate-rich byproducts are being explored to develop new AAMs.

Coal gangue (CG), a byproduct of coal mining, accounts for 10–25% of total coal production [21]. CG is one of the largest solid wastes in India, Australia, South Africa, and China [22]. China’s stockpile of CG is more than 7 billion tons; it occupies land resources and threatens the environment [23]. Published studies indicate that CG’s chemical composition is Al_2_O_3_ and SiO_2_; therefore, it has potential as a precursor for AAMs. Zhang et al. [24] revealed that 800 °C was optimal for forming metakaolin in calcined CG; however, the mechanical properties of the obtained AAMs decreased when the temperature reached 900 °C. Li et al. [21] explored the effects of CG particle size on the compressive strength of AAMs. Their results showed that 200 mesh CG achieved the necessary strength. In addition, Wang et al. [25] investigated alkali activator effects on the microstructures and mechanical properties of AAMs prepared from CG. They concluded that the maximum compressive strength of CG-based AAMs was 24.75 MPa, which required a Na_2_SiO_3_/NaOH ratio of 2.0 and a liquid/solid ratio of 0.50. Other research [25,26] indicates that the poor mechanical characteristics of CG-based AAMs are due to low activity, suggesting that improving the strength of CG-based AAMs is required.

Combining two or more precursors is an effective method for enhancing the mechanical performance of AAMs [27]. For example, Deb et al. [28] investigated AAMs with varying GGBFS/FA ratios (0%, 10%, and 20%). Their results showed that the maximum compressive strength (51 MPa) was achieved when the precursors were synthesized with 80% FA and 20% GGBFS. Bernal [29] prepared MK/GGBFS-based AAMs with a compressive strength of approximately 45 MPa (50% GGBFS) and 20 MPa (0% GGBFS). Additionally, Venkatesan and Pazhani [30] demonstrated that incorporating 10% rice husk ash (RHA) into GGBFS-based AAMs resulted in a compressive strength greater than 72.3 MPa. According to these studies, GGBFS (Ca-containing) AAM gels composed of calcium silicate hydrate (C-(A)-S-H) enhanced compressive strength [31], whereas Ca-free mineral-reaction gels (i.e., FA, MK, and RHA) composed of N-(A)-S-H decreased autogenous shrinkage [32].

Furthermore, the compressive strength of AAMs is significantly influenced by alkali activators such as the silicate modulus (SiO_2_/Na_2_O) and alkalinity (Na_2_O content) of the solution [33,34,35,36,37,38]. Pelisser et al. [34] found that a silicate modulus of 1.6 was beneficial for the mechanical properties of MK-based AAMs. However, Thaarrini et al. [35] recommended that a silicate modulus of 1.0 would achieve a higher compressive strength of FA-based AAMs, which differs from Pelisser et al.’s result of 1.6. Wang et al. [37] observed that the gel formation of MK-based AAMs increased as the NaOH solution concentrations (4–12 mol/L) (i.e., alkalinity) increased. Zhang et al. [38] found that the optimum NaOH concentration was 10 mol/L for FA-based AAMs and 12.5 mol/L for MK-based AAMs [33]. These findings highlight that the alkali activators’ optimum silicate modulus and alkalinity are distinctive for different precursors.

Therefore, the blending of precursors or alkali activation can promote the mechanical properties of AAMs. This study used a precursor of CG blended with GGBFS, and a mixture of Na_2_SiO_3_ and NaOH solutions served as the alkali activator. The research investigated the influence of the GGBFS/CG ratio, alkalinity (Na_2_O content), and alkali activator modulus (SiO_2_/Na_2_O) on the mechanical performance and microstructure of CG/GGBFS-based AAMs. The mineral composition, microstructure, and mechanical properties of AAMs were analyzed via X-ray diffraction (XRD), Fourier transform infrared (FTIR) spectroscopy, scanning electron microscopy (SEM), and energy-dispersive X-ray (EDX) spectroscopy. Additionally, mercury intrusion porosimetry (MIP) is employed to detect the pore characteristics and parameters of the AAMs. This study aimed to provide experimental and theoretical bases for the large-scale utilization of AAMs in construction.

## 2. Materials and Methods

### 2.1. Materials

The CG used in this study was sourced from Zhengzhou, Henan Province, China. Before use, it was subjected to calcination at 700 °C for 3 h. The GGBFS was procured from Nanyang, Henan Province, China.

**Figure 1:**
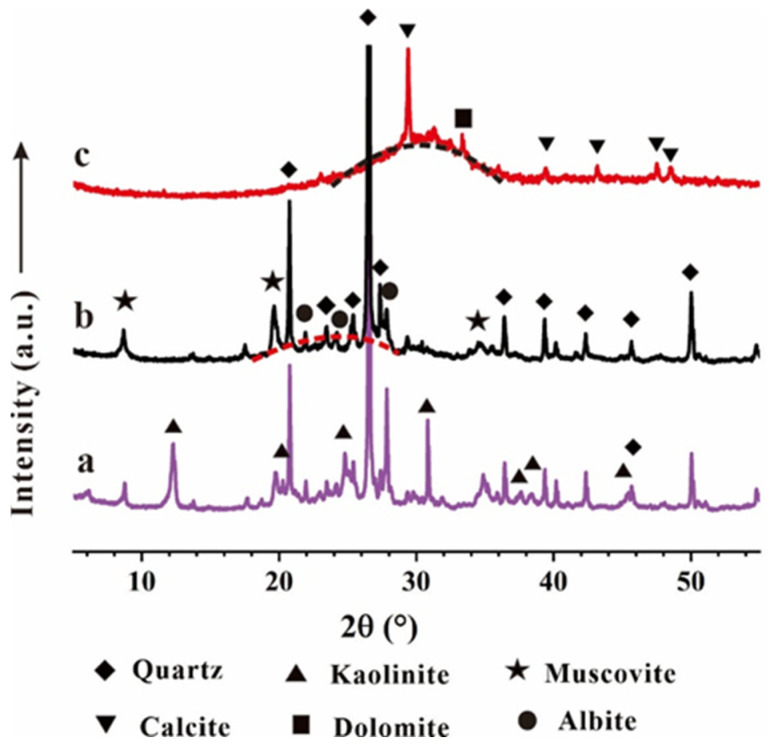
The XRD patterns of (**a**) Raw CG, (**b**) Calcined CG, and (**c**) Raw GGBFS.

Table 1 shows the chemical compositions of the CG and GGBFS, which were determined via X-ray fluorescence. The CG was rich in SiO_2_ and Al_2_O_3_, constituting 86.12 wt% of the total CG composition. The XRD patterns (Figure 1a) showed that quartz, kaolinite, and muscovite were the main minerals in the raw CG. Following the calcination process, the reflections of kaolinite disappeared, and the calcined CG exhibited a broad diffraction band centered at approximately 23.7° (2θ) (Figure 1b), suggesting the transformation of kaolinite into an amorphous phase, while quartz was preserved in the calcined CG due to its thermal stability. The GGBFS primarily contained CaO, SiO_2_, and Al_2_O_3_, which totaled 86.11 wt% of the whole composition. The XRD pattern of GGBFS (Figure 1c) featured a broad diffraction band centered at approximately 31.5° (2θ), indicating the GGBFS was predominantly amorphous. Calcite and dolomite were identified in the GGBFS.

**Figure 2:**
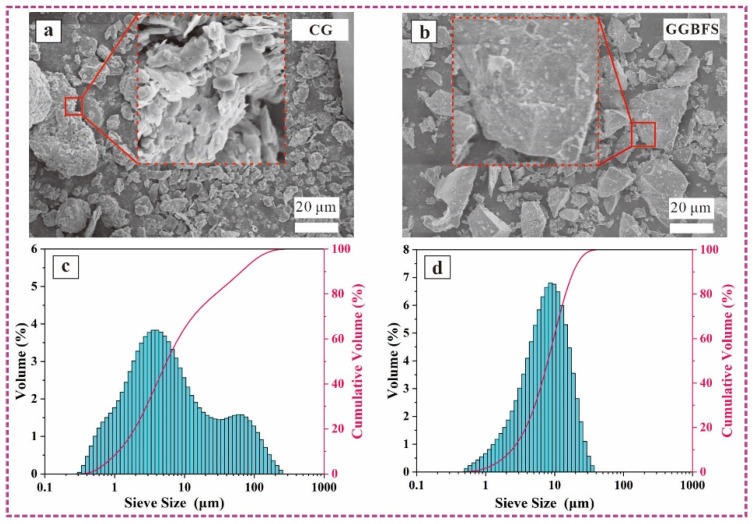
SEM images of CG (**a**) and GGBFS (**b**); and particle size distributions of CG (**c**) and GGBFS (**d**).

Figure 2 shows the SEM images and particle size distributions of the calcined CG and GGBFS. The CG possesses a loose surface morphology and porous structure (Figure 2a), and the GGBFS exhibits a smooth surface morphology and angular shape (Figure 2b). The median particle size (d_50_) of the CG and GGBFS are 8.59 and 10.29 μm, respectively.

The materials used as alkali activators were composed of sodium silicate solution (SiO_2_ 27.3 wt%, Na_2_O 8.5 wt%, and H_2_O 64.2 wt%) and sodium hydroxide particles (purity ≥96%). Distilled water was used for adjusting alkalinity. All chemical agents were obtained from laboratory and commercial vendors.

### 2.2. AAMs Preparation

**Figure 3:**
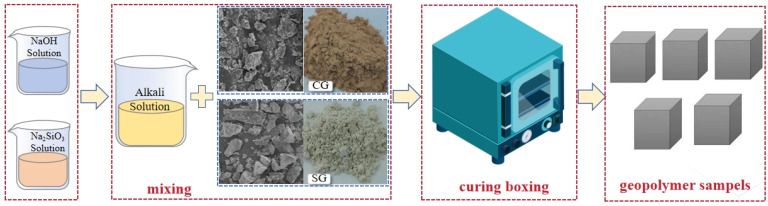
Preparation process of CG-based AAMs.

The preparation process is illustrated in Figure 3, and the specific mixing ratio of CG/GGBFS-based AAMs is detailed in Table 2. The AAMs were prepared by adding NaOH and Na_2_SiO_3_ to distilled water to formulate the alkali activators, and the precursors (calcined CG and GGBFS) were mixed for 2 min in a planetary mixer. The alkali activators were then blended with the precursors for 5 min to create a uniform slurry paste. The paste was subsequently poured into silica molds and vibrated on an electric vibration table for 90 s to eliminate the remaining bubbles. A thin polyethylene film was applied to cover all samples. Following this, the samples were cured in a controlled environment at 60 °C for 24 h, unmolded, and left at ambient temperature (~25 °C) at 3, 7, and 28 d before conducting the test.

The prepared AAMs were labeled S_X_C_Y_-W-Z, where S and C represent the raw precursors of GGBFS and CG; X and Y represent the mass ratios of GGBFS and CG; W indicates the Na_2_O content (mass ratio of Na_2_O); and Z indicates the alkali modulus (SiO_2_/Na_2_O) of the alkali activator. The liquid/solid ratio was 0.55. For example, S_1_C_1_-7-1.3 signified the specimens were fabricated with a mass ratio of GGBFS/CG of 1:1, and the Na_2_O content and alkali modulus were 7 wt% and 1.3, respectively.

### 2.3. Methods

#### 2.3.1. Physical Properties Tests

The fluidity of AAM paste was determined using a shortened conical apparatus (Cangzhou, China). The setting times of AAM paste were determined using a Vicat apparatus (Shanghai, China). The compressive strength of AAM samples was determined using an STS100K testing machine (Xiamen, China). Three samples were tested, and the average results were considered the final compressive strength.

#### 2.3.2. Microscopic Properties Tests

XRD patterns of the samples (raw materials and AAMs) were recorded on a D8 Advance diffractometer (Bruker, Mannheim, Germany) using Cu Kα radiation. The diffractometer was operated at 40 kV and 30 mA, and the scanning range was 3–70° (2θ) with a scanning speed of 3°/min. FTIR spectroscopy of AAM samples was recorded on a Nicolet IS 50 spectrometer (Thermo Fisher, Waltham, MA, USA). The spectra ranged from 400 to 4000 cm^−1^ with a resolution of 4 cm^−1^ and 64 scans. SEM images and EDX results of the samples (raw materials and AAMs) were obtained using an S-3400N-II instrument (Hitachi, Tokyo, Japan). The acceleration voltage was 35 kV, and the current was 10 mA. The MIP of AAM samples was measured using an AutoPore IV 9510 porosimeter (Micromeritics, Norcross, GA, USA). The intrusion pressures ranged from 0.5 to 33,000 psi with a contact angle of 130° and a surface tension of 485 dynes/cm.

## 3. Results

### 3.1. Setting Time and Compressive Strength

#### 3.1.1. Fluidity

The fluidity of the freshly mixed AAM pastes is shown in Table 2. The paste fluidity results changed from 107.6 to 167.7 mm as the mixture varied and was notably affected by the GGBFS content. For example, compared to C-7-1.3, the fluidity of pastes with GGBFS contents of 20%, 40%, 50%, 60%, 80%, and 100% increased by 9.76%, 20.17%, 25.74%, 32.71%, 43.49%, and 55.86%, respectively. This was attributed to the following: (1) CG has a more porous structure that can absorb free water; and (2) The smaller particle size of CG, inducing a larger specific surface area compared to GGBFS (Figure 2), increases the available water for the AAM paste, resulting in increased fluidity when CG is substituted with GGBFS. Kramar et al. [39] found that the fluidity of slag-based mortar was higher than that of FA (180 vs. 152 mm). However, the paste fluidity of the S_1_C_1_-W-1.3 samples was not altered (around 135.3 ± 3 mm). With changes in Na_2_O content from 6% to 9%, the fluidity increased from 133.2 to 138.2 mm. Similar changes were observed for the S_1_C_1_-7-Z samples; the fluidity changed from 127.3 to 139.5 mm as the SiO_2_/Na_2_O modulus increased from 0.9 to 1.5, respectively. This indicates that the Na_2_O content and modulus of the alkali activator were insensitive to the AAM paste fluidity.

#### 3.1.2. Setting Time

**Figure 4:**
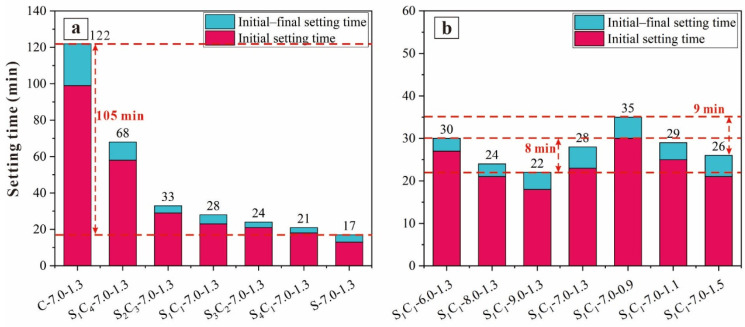
Setting times of AAMs: (**a**) S_X_C_Y_-7.0-1.3, and (**b**) S_1_C_1_-W-1.3 and S_1_C_1_-7.0-Z.

Figure 4 illustrates the setting times of the AAM pastes. The setting time was closely related to the geopolymerization rate, which was influenced by either the precursor materials or alkali activators. The final setting times of the AAM pastes appeared to be shortened, indicating that curing the samples in the controlled environment (60 °C) accelerated the geopolymerization process.

The setting times of the AAM pastes decreased as the GGBFS content increased, impacting both the initial and final setting times. Compared to the C-7.0-1.3 sample, the final setting times of samples with 100% GGBFS content (S-7.0-1.3) were significantly shortened, with an 86.1% reduction. This can be attributed to the higher reactivity of GGBFS; the reaction of the AAM paste increased when CG was replaced with GGBFS.

With increasing Na_2_O content, the setting time was slightly reduced (from 30 to 22 min), indicating that higher alkalinity (Na_2_O concentration) promoted the dissolution of Al and Si ions, thereby accelerating the geopolymerization process. However, with a higher alkali modulus (SiO_2_/Na_2_O), both the initial and final setting times of the AAM pastes were shortened. For example, the final setting time of S_1_C_1_-7.0-0.9 was 35 min, while that of S_1_C_1_-7.0-1.5 was shortened to 26 min when the alkali modulus increased to 1.5. Studies have reported that the higher alkali modulus in the reaction is conducive to geopolymerization, resulting in more AAM pastes and shorter paste hardening times [40].

#### 3.1.3. Compressive Strength

**Figure 5:**
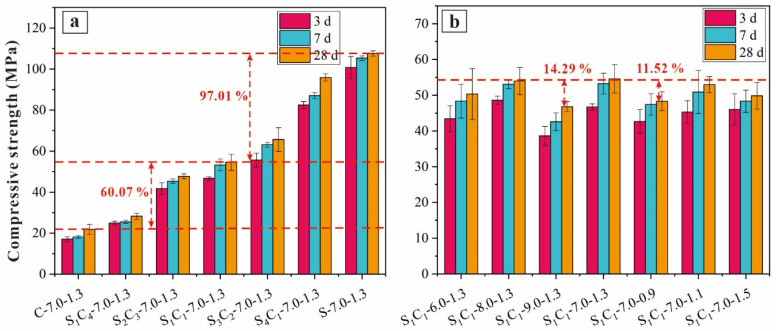
Compressive strength of AAM specimens (**a**) S_X_C_Y_-7.0-1.3, and (**b**) S_1_C_1_-W-1.3 and S_1_C_1_-7.0-Z.

Figure 5 illustrates the compressive strengths of the AAM specimens at various curing ages (i.e., 3 d, 7 d, and 28 d). The strength of the specimens exhibited a gradual increase throughout the curing period, indicating a continuous ongoing process of geopolymerization. Notably, there was a rapid increase in compressive strength during the early stages, especially by day 3. The compressive strength by day 3 was about 75% of that of the 28 d specimens, and by day 7, the compressive strength of the specimens was about 85% of that of the 28 d specimens. This was attributed to two factors: (1) more soluble silicates participated in the reactions during the initial stages of AAM curing, and (2) an elevation in curing temperature accelerated the geopolymerization rate.

Furthermore, with an increase in GGBFS content, the compressive strength of the AAM showed a significant improvement. The 28 d compressive strength of S-7.0-1.3 (100% GGBFS content) was 393.34% higher than that of C-7.0-1.3 (100% CG content). When the specimens had a CG/GGBFS ratio of 1:1, the compressive strength of S_1_C_1_-7.0-1.3 reached 54.59 MPa, equivalent to that of grade 52.5 OPC.

Moreover, an increase in the Na_2_O mass ratio from 6% to 8% initially improved compressive strength, followed by a decline in strength as the ratio increased to 9% (S_1_C_1_-9.0-1.3). Compared to S_1_C_1_-6.0-1.3, the compressive strengths of S_1_C_1_-7.0-1.3 and S_1_C_1_-8.0-1.3 increased by 8.44% and 7.21%, respectively, indicating that a higher Na_2_O mass ratio was beneficial for generating more paste gels and achieving higher compressive strength through enhanced dissolution of GGBFS and CG particles [41]. Increasing the Na_2_O mass ratio to 9% had a notable impact on the compressive strength of the AAMs. This was for two reasons: (1) inadequate alkalinity at low Na_2_O concentrations hindered polymerization; and (2) higher Na_2_O concentrations led to more precursors rapidly reacting with alkali activators, resulting in geopolymer gels forming on precursor surfaces and limiting their participation in polymerization.

With a rise in the alkali modulus, the compressive strength of the AAM specimens was only slightly affected. As the alkali modulus changed from 0.9 to 1.3, the compressive strength increased by 11.52%. However, the S_1_C_1_-7.0-1.5 specimens, with a modulus of 1.5, showed a lower compressive strength (49.85 MPa) than that of the S_1_C_1_-7.0-1.3 specimens (54.59 MPa), a decrease of 8.68%. This finding was contrasted with previous research. Guo et al. found that the compressive strength of 28 d FA-based AAMs significantly increased (from 17.1 to 59.3 MPa) as the SiO_2_/Na_2_O modulus changed from 1.0 to 1.5 [36].

### 3.2. Geopolymer Structure

#### 3.2.1. XRD Analysis

**Figure 6:**
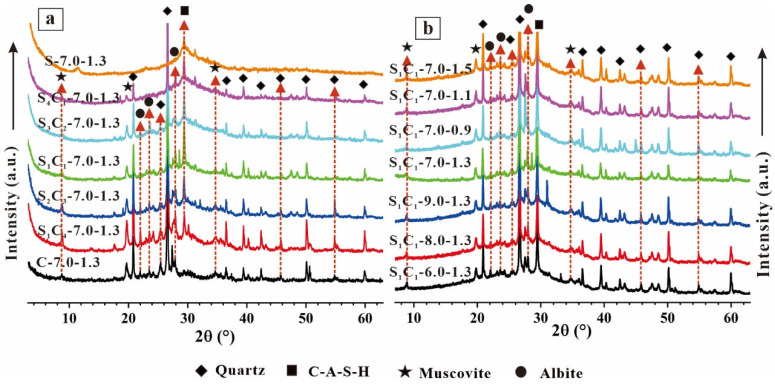
XRD patterns of AAM specimens (**a**) S_X_C_Y_-7.0-1.3, and (**b**) S_1_C_1_-W-1.3 and S_1_C_1_-7.0-Z.

Figure 6 illustrates the XRD patterns of the AAM specimens. There is a broad reflection within the 18°–40° (2θ), with a prominent peak at ~29° (2θ). Compared to the C-7.0-1.3 specimens, the patterns of the AAM specimens shifted toward a higher angle of ~30° (2θ) due to the inclusion of GGBFS. This indicates the presence of amorphous phases, which primarily consist of sodium aluminate silicate hydrate (N-A-S-H) and calcium aluminate silicate hydrate (C-A-S-H) [42,43,44]. He et al. [44] identified that the N-A-S-H gel was the product of low-calcium materials, and the C-A-S-H gel was predominant in rich-calcium precursors. Additionally, minerals like quartz and muscovite were detected in the specimens, suggesting that CG was insufficient for the geopolymerization reaction [45].

The intensity of the C-A-S-H crystalline phase increased with higher GGBFS content due to the reaction between the GGBFS and alkali activators [46,47]. However, increasing the Na_2_O mass ratio from 6% to 8% did not significantly impact mineral composition (Figure 6b). Compared to S_1_C_1_-6.0-1.3, the C-A-S-H diffraction peak of S_1_C_1_-8.0-1.3 was enhanced, indicating that more geopolymerization gels generated at higher alkalinity, resulting in the higher compressive strength of S_1_C_1_-8.0-1.3.

Varying the SiO_2_/Na_2_O modulus of the alkali solution had minimal effect on geopolymers’ compound synthesis. Compared to S_1_C_1_-7.0-0.9, the XRD patterns of S_1_C_1_-7.0-1.3 and S_1_C_1_-7.0-1.5 showed a lower intensity of the quartz diffraction peak, suggesting the greater consumption of quartz crystals in S_1_C_1_-7.0-1.3 and S_1_C_1_-7.0-1.5. This confirms that a higher SiO_2_/Na_2_O modulus promoted geopolymerization.

#### 3.2.2. FTIR Spectroscopy

**Figure 7:**
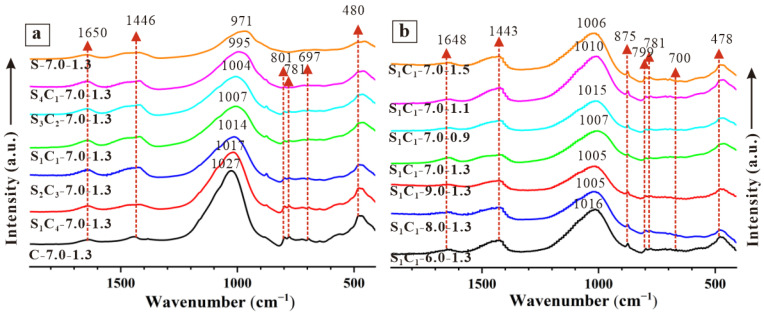
FTIR spectra of AAM specimens (**a**) S_X_C_Y_-7.0-1.3, and (**b**) S_1_C_1_-W-1.3 and S_1_C_1_-7.0-Z.

Figure 7 displays the FTIR spectra of the AAM specimens between 400 and 1800 cm^−1^, while Table 3 provides the vibrational modes of FTIR band assignments for the selected samples. In Figure 7a, a broad band between 800 and 1200 cm^−1^ was observed and shifted, indicating the presence and alteration of amorphous products [48,49]. With an increase in GGBFS content, the spectra of the specimens shifted towards lower wavenumbers. For example, compared to C-7.0-1.3, the center of the S_1_C_1_-7.0-1.3 and S-7.0-1.3 broad bands moved from 1027 to 1007 and 971 cm^−1^, respectively. Research [50] indicated that the N-A-S-H gel is primarily associated with 1030 cm^−1^, while the C-A-S-H gel is mainly focused on 940 cm^−1^. This suggests that the components of the AAM specimens were transformed from N-A-S-H to C-A-S-H as the GGBFS content increased.

In Figure 7b, as the Na_2_O mass ratio increased, the characteristic bands decreased from 1016 cm^−1^ (S_1_C_1_-6.0-1.3) to 1005 cm^−1^ (S_1_C_1_-9.0-1.3). Additionally, with an increase in SiO_2_/Na_2_O modulus from 0.9 to 1.5, the center of the broad band decreases from 1015 cm^−1^ (S_1_C_1_-7.0-0.9) to 1006 cm^−1^ (S_1_C_1_-7.0-1.5). These minimal changes in FTIR peaks indicated limited impacts on the composition of the AAM specimens within the activated system.

#### 3.2.3. SEM/EDX Results

**Figure 8:**
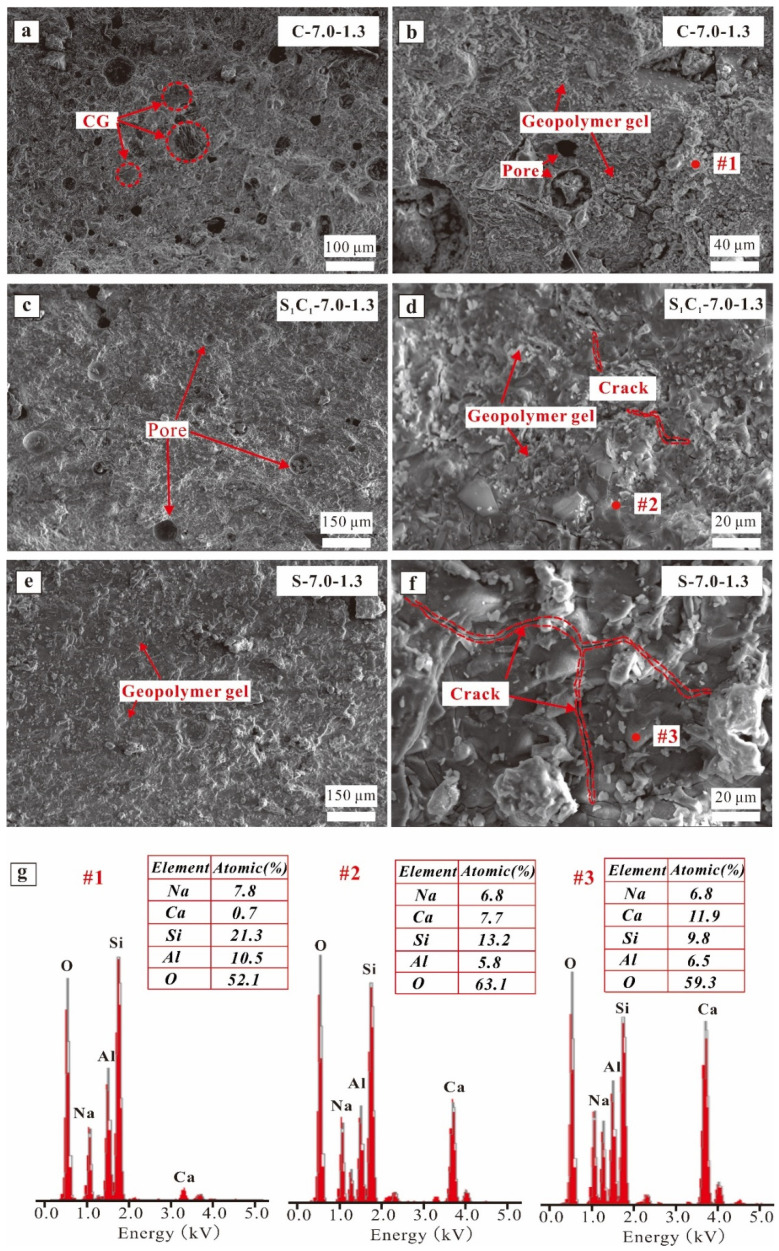
SEM images of AAM specimens: (**a**,**b**) C-7.0-1.3, (**c**,**d**) S_1_C_1_-7.0-1.3, and (**e**,**f**) S-7.0-1.3; (**g**) EDX results of spots highlighted in images (**b**,**d**,**f**).

**Figure 9:**
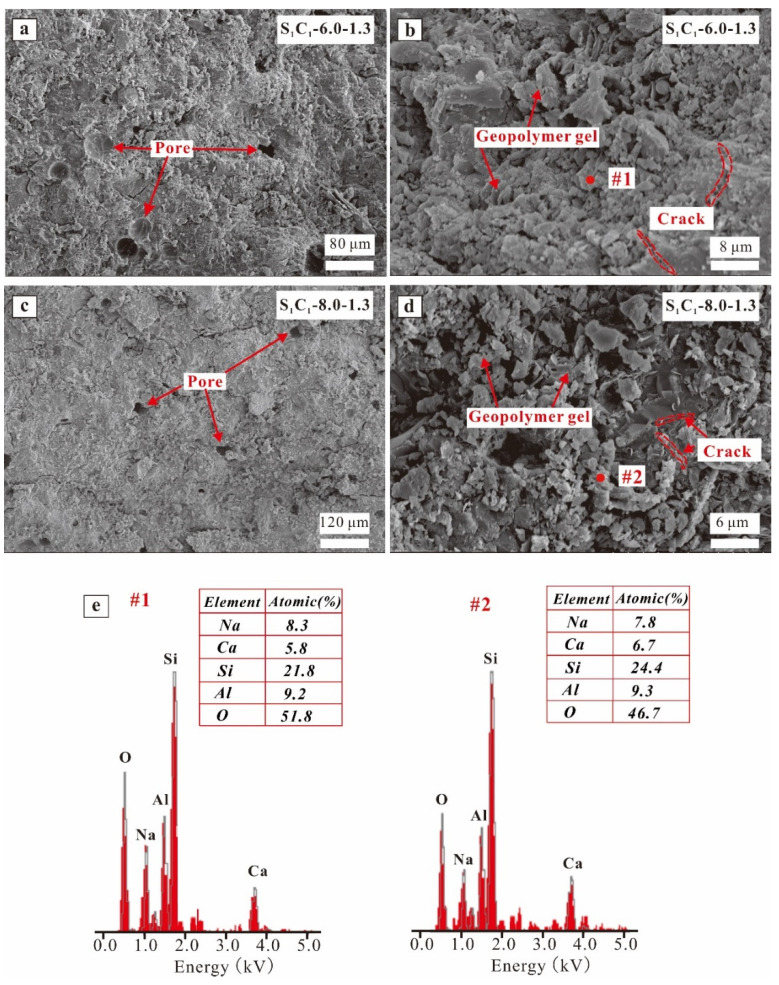
SEM images of AAM specimens: (**a**,**b**) S_1_C_1_-0.6-1.3, (**c**,**d**) S_1_C_1_-0.8-1.3, and (**e**) EDX results of spots highlighted in images (**b**,**d**).

**Figure 10:**
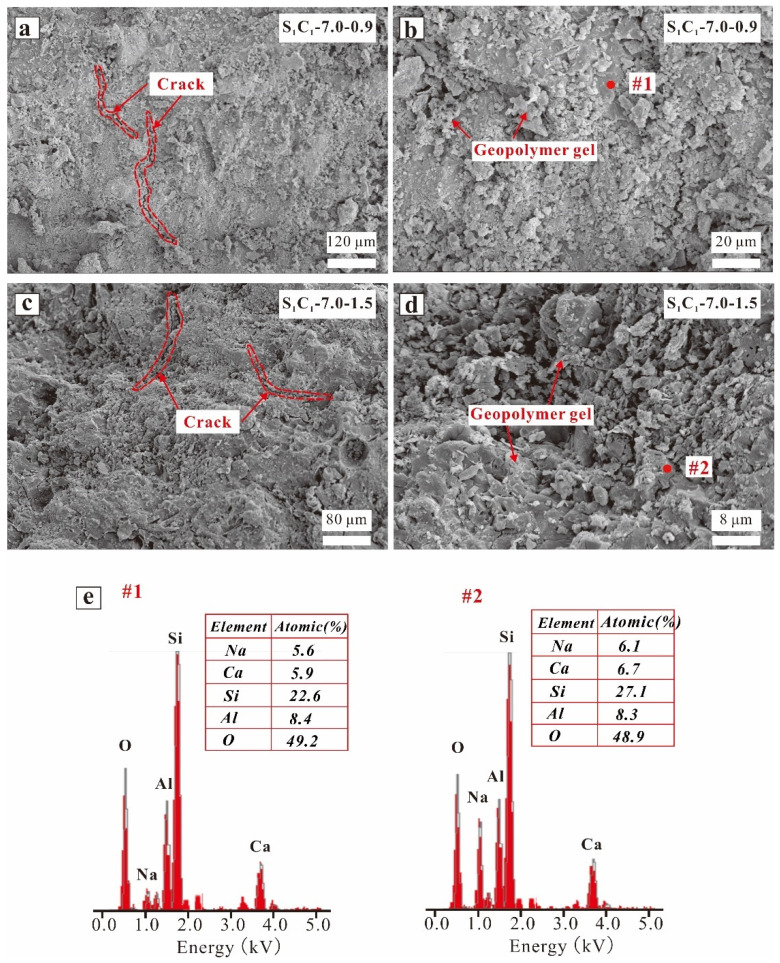
SEM images of AAM specimens: (**a**,**b**) S_1_C_1_-7.0-0.9, (**c**,**d**) S_1_C_1_-7.0-Z, and (**e**) EDX results of spots highlighted in images (**b**,**d**).

Figure 8, Figure 9 and Figure 10 illustrate the SEM/EDX findings of the AAM samples. The microstructures of the specimens with various GGBFS contents (i.e., 0%, 50%, and 100%) are examined in Figure 8a–g. In Figure 8a, the C-7.0-1.3 specimens possess a loose microstructure that coexists with isolated particles in the matrix. As the GGBFS content increased, the microstructure of the S_1_C_1_-7.0-1.3 and S-7.0-1.3 specimens became more compact (Figure 8c,e), and there were fewer smaller pores in the stone body. This suggested that more AAM gels (C-(N)-A-S-H) were produced as the GGBFS content increased, leading to enhanced compressive strength. The high-magnification SEM images (Figure 8d,f) showed microcracks in the bodies of the S_1_C_1_-7.0-1.3 and S-7.0-1.3 specimens. Research [61,62,63] revealed that the shrinkage value of the AAMs increased with the addition of GGBFS, contributing to the formation of more microcracks. The EDX analysis (Figure 8g, spot 1) revealed that the C-7.0-1.3 specimens were primarily composed of N-A-S-H, while the S_1_C_1_-7.0-1.3 and S-7.0-1.3 specimens (Figure 8g, spots 2 and 3) contained C-(N)-A-S-H, which aligns with the elemental analysis. Additionally, the analyzed AAM samples exhibited varying Ca/Si ratios. For example, the Ca/Si ratio of C-7.0-1.3 was 0.03, considerably lower than that of S_1_C_1_-7.0-1.3 (0.59) and S-7.0-1.3 (1.27). This result suggests that the formation of more C-A-S-H gel was generated with increasing GGBFS content. Temuujin et al. [31] demonstrated that the more C-A-S-H gel generated, the higher the compressive strength achieved for FA-based AAMs.

As the Na_2_O mass ratio increased from 6% to 8%, there was no apparent difference observed between the S_1_C_1_-0.6-1.3 and S_1_C_1_-0.8-1.3 specimens, with some pores being evident in the matrix of their compact microstructure (Figure 9a,c). The high-magnification SEM images showed an increase in AAM gels generated with greater Na_2_O content; however, some microcracks occurred in the stone body (Figure 9b,d). The EDX results, as depicted in Figure 9e (spots 1, and 2), demonstrated that the Ca/Si ratios were similar between the S_1_C_1_-0.6-1.3 and S_1_C_1_-0.8-1.3 (0.26 vs. 0.27); the (Ca + Na)/(Si + Al) ratios of the S_1_C_1_-0.6-1.3 and S_1_C_1_-0.8-1.3 were 0.45 and 0.43, respectively. Furthermore, the lower Ca/Si ratio in S_1_C_1_-0.6-1.3 resulted in a decrease in compressive strength.

Figure 10 illustrates the SEM images and EDX findings of the S_1_C_1_-7.0-0.9 and S_1_C_1_-7.0-1.5 specimens, with both exhibiting compact morphology (Figure 10a,c). In addition, the high-magnification SEM images showed that less AAM gel was generated in the S_1_C_1_-7.0-0.9 specimens due to the incomplete reaction of the low soluble silicate content. The EDX results, as depicted in Figure 10e (spots 1 and 2), demonstrate that the Ca/Si ratios of the S_1_C_1_-7.0-0.9 and S_1_C_1_-7.0-1.5 (0.26 vs. 0.25) specimens were similar, and the (Ca + Na)/(Si + Al) ratios of the S_1_C_1_-7.0-0.9 and S_1_C_1_-7.0-1.5 were 0.37 and 0.36, respectively, suggesting that more Si atoms were involved in the S_1_C_1_-7.0-1.5 reaction.

#### 3.2.4. MIP Results

**Figure 11:**
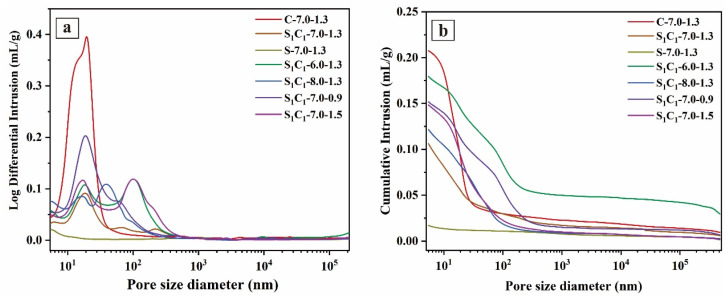
MIP results of AAM specimens: (**a**) log differential intrusion; (**b**) cumulative intrusion.

Figure 11 displays the distribution of pore size in the AAM specimens, and the pore parameters are shown in Table 4, which were analyzed by MIP. In Figure 11a, the samples exhibited a predominant pore size range of 10 to 200 nm. They were primarily medium capillary pores (10–100 nm) and large capillary pores (100–1000 nm), with large capillary pores having a negative impact on the mechanical properties of the AAMs [64].

With 100% CG, the average pore size of C-7.0-1.3 specimens was 35.70 nm, and the porosity was 31.74%. As the GGBFS content increased, the pore size and porosity of the specimens decreased. For example, the average pore sizes of S_1_C_1_-7.0-1.3 (50% GGBFS content) and S-7.0-1.3 (100% GGBFS content) were 22.84 and 17.06 nm, respectively, and the porosity decreased by 42.1% and 89.4%, respectively. Figure 11b illustrates the decline in the cumulative pore volume of the AAM specimens with added GGBFS, showing a higher cumulative intrusion for C-7.0-1.3 (0.208 mL/g) compared to that for S-7.0-1.3 (0.017 mL/g). This suggests that incorporating GGBFS boosts the reactivity of raw precursors, resulting in the generation of more AAM gels and the compact structures of the specimens.

Additionally, the S_1_C_1_-6.0-1.3 specimens exhibited a critical pore size of 31.71 mm, which was greater than that of S_1_C_1_-8.0-1.3. This indicated that increasing Na_2_O content promotes hydrolysis of the aluminosilicate precursors and improves the compact structure of the specimens. These findings are aligned with the compressive strengths of the samples. Porosity analysis in Table 4 revealed that S_1_C_1_-6.0-1.3 had a porosity of 27.19%; therefore, the greater pore size induced the inferior compressive strength of S_1_C_1_-6.0-1.3 compared to S_1_C_1_-8.0-1.3 (Figure 5b). Fan et al. [65] emphasized the impact of pore dimensions on the mechanical characteristics of cementitious substances.

Furthermore, the porosity of the AAM specimens remained unchanged (~24.50%) as the SiO_2_/Na_2_O modulus varied, while the average pore size of S_1_C_1_-7.0-0.9 was larger (29.07 mm) than that of S_1_C_1_-7.0-1.5 (21.28 mm). This suggests that an insufficient number of Si atoms participate in the S_1_C_1_-7.0-0.9 reactions, resulting in a larger pore size (Table 4), consistent with the change in compressive strength.

## 4. Discussion

The research determined that the compressive strength and microstructure of CG/GGBFS-based AAMs were influenced by GGBFS and alkali activator contents. As the GGBFS content increased from 0% to 50% (or 100%), more AAM gels (C-(A)-S-H) were generated, forming a three-dimensional network and resulting in a higher compressive strength [66]. For example, the compressive strength was 47.72 MPa for S_2_C_3_-7.0-1.3 (40% GGBFS content), and that of S_1_C_1_-7.0-1.3 (50% GGBFS content) was 54.59 MPa. This is favorable for the preparation of AAM concrete as an alternative to OPC materials. Additionally, as the Na_2_O mass ratio changed from 6% to 9%, the compressive strength increased by 8.44%, which was compared to that of S_1_C_1_-6.0-1.3. The variation of the SiO_2_/Na_2_O modulus from 0.9 to 1.5 only slightly affected the strength of the AAM specimens (48.3 vs. 54.59 MPa). This suggests that the compressive strength of CG/GGBFS-based AAMs is mainly dependent on the GGBFS content. A Na_2_O mass ratio of 7% and a SiO_2_/Na_2_O modulus of 1.3 were the most beneficial for generating AAM gels.

Furthermore, the setting times of the CG/GGBFS-based AAMs were faster due to the high reactivity of GGBFS. Several researchers have explored how a retarder admixture prolongs the setting times of AAM systems. For example, Wang et al. [67] found that zinc nitrate and sodium gluconate were excellent retarders for slag-based AAMs. Cong et al. [68] modified the properties of FA-based AAMs by adding borate. Both Kalina [69] and Gong [70] explored how sodium phosphate influences the hydration process of red mud and slag-based AAMs. Their findings indicated that sodium phosphate effectively prolonged the hydration and the setting time for AAMs. In related work, Brough et al. [71] reported that incorporating 0.5 wt% malic acid extended the initial setting time, which ranged from 4 to 20 h. The literature records effective retarders for controlling the setting times of AAMs, but fewer studies have discussed the effects of existing retarders on CG/GGBFS-based AAMs; this remains an investigation for future work.

## 5. Conclusions

This study investigated the effects of the GGBFS/CG ratio, alkalinity (Na_2_O content), and alkali modulus (SiO_2_/Na_2_O) on the mechanical properties and microstructures of CG/GGBFS-based AAMs. The results of a series of spectroscopic and microscopic tests led to the following conclusions:

The addition of GGBFS significantly influenced the compressive strength and microstructure of CG/GGBFS-based AAMs. Higher GGBFS content led to more AAM gels (C-(A)-S-H) generated, resulting in the fluidity of the AAM paste increasing. Alkali activators (Na_2_O and SiO_2_/Na_2_O contents) had only a slight influence on the chemical composition and microstructure of the AAM.

The compressive strengths of S_2_C_3_-7.0-1.3 and S_1_C_1_-7.0-1.3 were 47.72 MPa and 54.59 MPa, respectively, which favors the use of AAM concrete for OPC materials. However, by increasing the Na_2_O mass ratio from 6% to 9%, more precursor materials (CG and GGBFS) participated in the depolymerization reactions, resulting in the structure of the AAMs becoming more compact. The porosities of S_2_C_3_-7.0-1.3 and S_1_C_1_-7.0-1.3 decreased from 27.19% to 20.58%, and their compressive strengths increased by 8.44% compared to S_1_C_1_-6.0-1.3. As the alkali modulus changed, specimens with a lower alkali modulus (S_1_C_1_-7.0-0.9) exhibited a loose microstructure, resulting in decreased compressive strength. The porosity of the AAM specimens was approximately 24.50%, and the compressive strengths of S_1_C_1_-7.0-0.9 and S_1_C_1_-7.0-1.5 were 48.3 MPa and 49.85 MPa, respectively.

The findings of this research provide theoretical and technical insights into the reuse of CG and GGBFS waste and the applications of CG- and GGBFS-based AAMs.

## Figures and Tables

**Table 1:** Main chemical composition of CG and GGBFS (wt%).

Chemical Composition	SiO_2_	Al_2_O_3_	Fe_2_O_3_	CaO	Na_2_O	MgO	K_2_O	TiO_2_	Others
CG	62.84	23.28	4.44	1.73	1.04	1.99	3.22	0.98	0.48
GGBFS	28.03	14.65	0.44	43.43	0.51	8.57	0.44	0.99	2.94

**Table 2:** Mixture proportions of CG/GGBFS-based AAMs.

Specimens	CG (wt%)	GGBFS (wt%)	Na_2_O (%)	Ms = (SiO_2_/Na_2_O)	Liquid/Solid	Fluidity (mm)
C-7.0-1.3	0	100	7.0	1.3	0.55	107.6
S_1_C_4_-7.0-1.3	20	80				118.1
S_2_C_3_-7.0-1.3	40	60				129.3
S_1_C_1_-7.0-1.3	50	50				135.3
S_3_C_2_-7.0-1.3	60	40				142.8
S_4_C_1_-7.0-1.3	80	20				154.4
S-7.0-1.3	100	0				167.7
S_1_C_1_-6.0-1.3	50	50	6.0	1.3		133.2
S_1_C_1_-8.0-1.3			8.0			137.1
S_1_C_1_-9.0-1.3			9.0			138.2
S_1_C_1_-7.0-0.9			7.0	0.9		127.3
S_1_C_1_-7.0-1.1				1.1		131.9
S_1_C_1_-7.0-1.5				1.3		139.5

**Table 3:** The vibrational modes of FTIR band assignments for the selected samples.

Wavenumber (cm^−1^)	Assignment	References
1650	Stretching vibration of O–H bonds	[51]
1445	Asymmetric stretching vibration of C–O bonds	[52]
1081-1010	In-plane stretching vibrations of Si–O bonds	[53,54]
875	Out-of-plane bending vibrations of C–O bonds	[55]
801	Symmetric stretching vibrations of Si–O bonds	[56]
780	Stretching vibration of Al–O bonds	[57]
697	Internal extension of the Si–O bond	[58]
480	Stretching vibration of Si–O–T bands	[59,60]

**Table 4:** The pore parameters for the AAM samples determined via MIP.

Samples	Total Pore Area (m^2^/g)	Average Pore Diameter (nm)	Porosity (%)
C-7.0-1.3	36.58	35.70	31.74
S_1_C_1_-7.0-1.3	24.89	22.84	18.38
S-7.0-1.3	7.64	17.06	3.36
S_1_C_1_-6.0-1.3	20.11	25.93	27.19
S_1_C_1_-8.0-1.3	22.84	21.29	20.58
S_1_C_1_-7.0-0.9	27.91	29.07	24.58
S_1_C_1_-7.0-1.5	20.88	21.28	24.42

## Data Availability

The original data will be made available upon requirement.
